# Loss of Fanconi anemia proteins causes a reliance on lysosomal exocytosis

**DOI:** 10.1038/s41419-025-08164-0

**Published:** 2025-11-04

**Authors:** Becky Xu Hua Fu, Albert Xu, Hua Li, Daniel E. Johnson, Jennifer R. Grandis, Luke A. Gilbert

**Affiliations:** 1 Arc Institute, 3181 Porter Drive, Palo Alto, CA 94304 USA; 2 Department of Urology, University of California, San Francisco, San Francisco, CA USA; 3 Department of Cell and Tissue Biology, University of California, San Francisco, San Francisco, CA USA; 4 Medical Scientist Training Program, University of California, San Francisco, San Francisco, CA 94158 USA; 5 Department of Otolaryngology-Head and Neck Surgery, University of California, San Francisco, San Francisco, CA USA; 6 University of California, San Francisco, Helen Diller Family Comprehensive Cancer Center, San Francisco, CA 13 CA USA; 7 Innovative Genomics Institute, University of California, Berkeley, Berkeley, CA USA

**Keywords:** Cell biology, Cancer, Molecular biology, Cell signalling, Stress signalling

## Abstract

Mutations in the Fanconi Anemia (FA) pathway lead to a rare genetic disease that increases risk of bone marrow failure, acute myeloid leukemia, and solid tumors. FA patients have a 500 to 800-fold increase in head and neck squamous cell carcinoma compared to the general population and the treatments for these malignancies are ineffective and limited due to the deficiency in DNA damage repair. Using unbiased CRISPR-interference screening, we found the loss of FA pathway function renders cells dependent on key exocytosis genes such as SNAP23. Further investigation revealed that loss of FA pathway function induced deficiencies in lysosomal health, dysregulation of autophagy and increased lysosomal exocytosis. The compromised cellular state caused by the loss of FA genes is accompanied by decreased lysosome abundance and increased lysosomal membrane permeabilization in cells. We found these signatures in vitro across multiple cell types and cell lines and in clinically relevant FA patient cancers. Our findings are the first to connect the FA pathway to lysosomal exocytosis and thus expands our understanding of FA as a disease and of induced dependencies in FA mutant cancers.

## Introduction

Fanconi anemia (FA) is a rare genetic disease that occurs in 1 in 300,000 live births. FA is caused by either inheriting biallelic germline loss-of-function mutations in one of the 22 genes (FANCA/C/D1/D2/E/F/G/I/J/L/M/N/O/P/Q/S/T/U/V/W) in the FA pathway or a monoallelic loss-of-function of RAD51 [1]. The 23 genes associated with FA are all involved in a DNA repair pathway that detects covalently linked DNA strands (inter-strand crosslinks: ICL) and mediates repair through homologous recombination. Due to the deficiency in DNA repair machinery, FA patients have an increased risk in bone marrow failure (BMF)/acute myeloid leukemia (AML). Although modern bone marrow transplant technology has allowed FA patients to overcome BMF and AML, FA patients have increased rates of solid tumors at remarkably young ages. Head and neck squamous cell carcinoma (HNSCC) are the most common solid tumor malignancy in FA patients with a frequency 500-fold to 800-fold higher in FA compared to the general population [2].

In addition, the inherent defect in DNA repair machinery in FA patients complicates treatment of FA patients’ cancers. The treatments include surgical removal and/or controlled low doses of chemotherapy, cross linking agents (cisplatin), and radiation. FA cancer patients usually suffer from severe radiation and chemotherapy toxicities even with low doses. Due to the limited options of treatment, the average life expectancy of FA patients is 20–30 years [3]. The dire reality of the health issues for people with FA highlights the importance of having a more holistic understanding of the biology of FA pathway and the implications of its absence.

The majority of studies that characterize FA pathway function have focused on the critical role that FA proteins play in the repair of ICLs and in genome maintenance. However, noncanonical roles, including stabilization of replication forks and regulation of cytokinesis, have also been established [4]. Additionally, functional genomics technologies have allowed for knowledge on even well studied pathways such as FA to be expanded. For example, a previous study that used unbiased genome-scale RNAi screens designed to identify genes that regulate autophagy unexpectedly identified FA genes. Further characterization of the identified FA genes showed that the FA pathway is involved in mitophagy [5, 6].

To further identify and characterize noncanonical functions and biology of the FA pathway, we performed genome-scale CRISPR-interference (CRISPRi) screens in isogenic FA pathway mutant (FANCD2) and wild-type (WT) cellular models to identify genetic dependencies induced by loss of the FA pathway activity. We unexpectedly found that genes related to lysosomal exocytosis are required for cell viability upon loss of FANCD2. Characterization of top hits such as SNAP23 and STX4, which are important for lysosomal exocytosis, led us to discover inherent lysosomal deficiencies in FA mutant cells. Our findings reveal an unexpected phenotype with the loss of FA pathway that advances our understanding of FA beyond a disease of DNA repair to include defects in quality control systems in lysosomal pathways that cause dysregulation of autophagy, which can render cells dependent on lysosomal exocytosis.

## Results

### Genome-scale CRISPRi screens identify lysosomal related genes as specific FA-deficient genetic dependencies

**Fig. 1:**
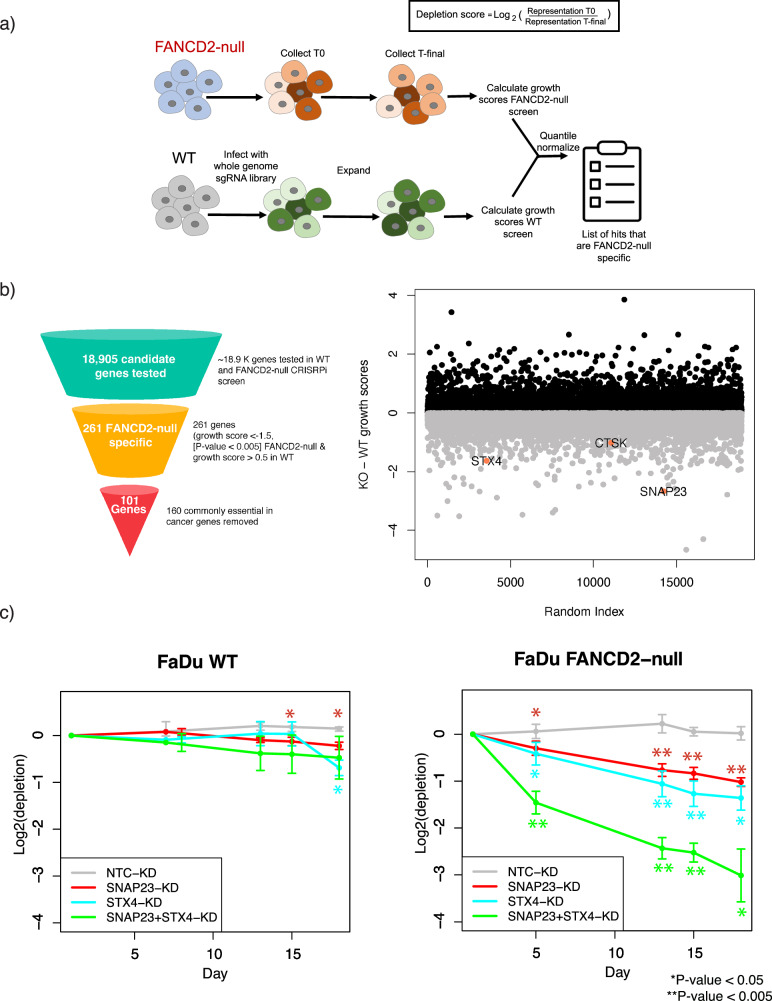
CRISPRi screen nominates lysosomal biology as an induced dependency upon loss of FANCD2. **a** A schematic of genome scale CRISPRi screens performed in FaDu WT and FANCD2-null cells. The depletion phenotypes from each screen were calculated, normalized, and compared between the WT and the mutant background to find candidate genes that were more depleted in the FANCD2-null background. **b** The funnel diagram depicts the filtering to narrow down candidates from the screen (left). To the right is a scatter plot of CRISPRi screening results comparing FANCD2-null to WT conditions. Our screens nominated 101 candidate genes that are conditional dependencies in the FANCD2-null but not in the WT background after filtering for commonly essential genes. Genes that were involved in lysosomal and lysosomal exocytosis biology are highlighted in pink. Normalized depletion scores from the FANCD2-null background were subtracted from WT and plotted against a random index to visualize the spread of the differences between depletion scores. **c** A graph showing a competitive growth-based assay analyzed over time to validate top FANCD2-conditionally essential CRISPRi screen hits in FaDu WT and FANCD2-null background. SNAP23 and STX4 were knocked down individually and together in both backgrounds and depletion was tracked over time (*n* = 3 as biological replicates; Mean ± STD, Unpaired two-tailed *t*-test was used to determine statistical significance).

To characterize the genetic dependencies of an FA-deficient versus WT genetic background, we used CRISPR-Cas9 to engineer a FA deficiency (FANCD2-null) in a well-characterized head and neck cancer cell line (FaDu) (Supplementary Fig. S1). The FaDu cell line was chosen because the Fanconi Cancer Foundation (FCF) has available open source FANCA knockout (FANCA-null) and FANCA-null rescued with transgene (FANCA-null + Tg) isogenic FaDu and UM-SCC-01 (another head neck cell line) cell lines that complement our study design and provide convenient independent validation. We further engineered the parental FaDu and FANCD2-null cell models to stably express CRISPRi (dCas9-KRAB) protein. We performed genome-scale CRISPRi screens targeting 18,905 protein coding genes in both FA mutant and WT FaDu CRISPRi cell lines and compared quantile normalized gene depletion scores between mutant and WT screen data to identify genes that are selectively required for cellular growth or survival in the FANCD2-null background (Fig. 1a, see Methods for details).

We defined FANCD2-null conditionally essential candidate hit genes as having a depletion score lower than −1.5 and a *p*-value < 0.005 in the FANCD2-null screen and little to no growth effect in the WT background (depletion score > −0.5). The comparison between isogenic screens identified 261 genes that were conditionally essential in the FANCD2-null vs WT background (Supplementary Table S1). Gene Ontology (GO) analysis using ShinyGO [7] of the 261 unfiltered candidate genes specific to FANCD2-null background identified predictable pathways for a FA mutant, including cell cycle and chromosome segregation (Supplementary Fig. S2). This gene list was then filtered by removing all genes annotated as common essential by the Cancer Dependency Map (DepMap) [8] (see Supplementary Table S2 for common essential gene list that was used). The final FANCD2 conditionally essential candidate gene list consisted of 101 candidate genes (Fig. 1b-left, Supplementary Table S3). GO analysis of the 101 candidate genes showed no significant enriched pathways. Upon examination of the molecular functions of FANCD2-specific candidates, we noted top candidate hit genes involved in lysosomal and lysosomal exocytosis biology. For example, the lysosomal and lysosomal exocytosis related genes Cathepsin K (CTSK), Synaptosome associated protein of 23 kDa (SNAP23) and Syntaxin 4 (STX4) are highlighted (Fig. 1b-right). Given that FA genes have been recently shown to play a key role in mitophagy, we hypothesized that these lysosome related hit genes which are FANCD2-null conditionally essential could be of considerable interest and point to how FA-deficiency broadly effects cell biology beyond canonical DNA repair defects. We confirmed that SNAP23-knockdown (KD) does not cause increase in DNA damage in FaDu FANCD2-null cells by quantification of protein levels of γH2AX and DNA fragmentation (TUNEL assay) (Supplementary Fig. S3a, b).

### Loss of FA causes an increase in lysosomal exocytosis and a reliance on lysosomal exocytosis

Lysosomes are important degradative organelles that play a crucial role in cellular homeostasis. Lysosomes not only contribute to clearance and recycling of cellular components but there has also been evidence to show that lysosomes and other organelles of autophagic and endo-lysosomal systems can be released for clearance and cell-to-cell communication and can play a role in plasma membrane repair through a process called lysosomal exocytosis [9, 10]. The process of lysosomal exocytosis involves the formation of a trans-SNARE complex made up of vesicle associated membrane protein 7 (VAMP 7), SNAP23, and STX4. The trans-SNARE complex allows for the docking and the fusion of the membranes of the lysosomes and endo-lysosomal vesicles to the plasma membrane which allows for the release of its contents [11]. Lysosomal exocytosis is one of the three known methods of cellular clearance (autophagosome/amphisome secretion, exosomes). Studies have shown that cells with lysosomal damage respond by using migratory autolysosomes to expel autolysosomes via exocytosis.

To confirm that loss of FA activity induces a cellular dependency on lysosomal exocytosis through SNAP23, we validated our CRISPRi screen results using a mixed competition growth assay (see Methods). We observed that SNAP23 repression in FANCD2-null genetic background shows a statistically significant increased depletion compared to WT with non-targeting control (NTC)-KD in FaDu cells, ~−1 vs ~0.14 depletion score, respectively (Fig. 1c). To understand if the SNAP23-KD inhibited growth or caused cell death, we measured the levels of Caspase 3/7 activation (CellEvent™ Caspase-3/7 Green Flow Cytometry). We found a statistically significant increase in Caspase 3/7 activity with the SNAP23-KD in both WT and FANCD2-null backgrounds (Supplementary Fig. S3c). In addition, we observed comparable depletion results upon knockdown of SNAP23 in a FANCA-null background versus WT background in Cal33 cells, another head neck cancer cell line (Supplementary Fig. S4). Because lysosomal exocytosis has previously been shown to require SNAP23 as well as syntaxin 4 (STX4) and VAMP7 [11], we also measured the phenotype of simultaneous knockdown of SNAP23 and STX4. This resulted in a significantly stronger negative growth phenotype relative to single gene knockdown of either SNAP23 or STX4 in the FANCD2-null background but not in the WT background in FaDu cells (Fig. 1c). These experiments validated our screen results and indicated that deficiency in the FA pathway induces a dependency involving lysosomal exocytosis. SNAP23 has been previously characterized to form docking neuronal SNARE complexes with SNAP25 that function in calcium triggered exocytosis [12–15]. Although SNAP25 is not a significant candidate in the screen and has no effect alone in knockdown validation experiments, the repression of SNAP23 and SNAP25 showed synergistic depletion in early timepoints compared to the single knockdowns in the FANCD2-null background (Supplementary Fig. S5). Together our results suggest that loss of FANCD2 function may render cells reliant on lysosomal exocytosis for survival.

SNAP23 and STX4 control various intracellular trafficking pathways that could affect viability. Although we cannot rule out viability effects of the loss of SNAP23 is not due to its role in general trafficking, an analysis of various trafficking and general autophagy genes (25/27 genes) in the FANCD2-null CRISPRi screening results show no significant effects in depletion score. Two genes had statistically significant depletion scores: ATG16L1(gene necessary for autophagy by regulating membrane trafficking [16]) had ~−0.96 depletion score while Rab5A (protein that regulates the movement of vesicles by facilitating internalization and fusion to early endosomes [17]) had a score of ~0.63 (Supplementary Fig. S6). The lack of effect of most general trafficking genes in the FANCD2-null background on the viability suggests that the SNAP23 knockdown phenotype we observe is unrelated to general trafficking.

**Fig. 2:**
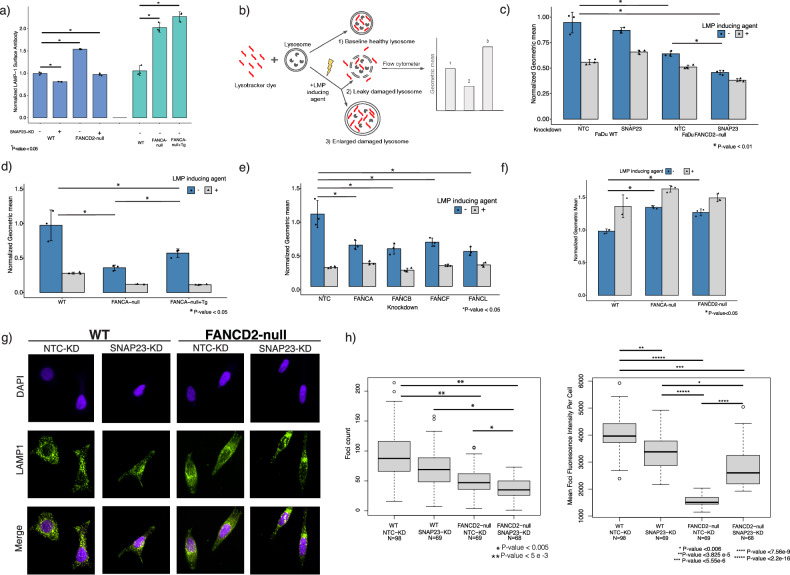
Loss of the FA pathway results in deficiencies in lysosomal health. **a** Lysosomal exocytosis was quantified using antibody binding surface LAMP-1. FaDu WT and FANCD2-null cells with and without SNAP23 repression and FA-deficient (FANCA-null/FANCA-null+Tg) cell lines were tested. LAMP-1 scars on the plasma membrane were quantified by flow analysis (*n* = 3 as biological replicates; unpaired *t*-test was used to determine statistical significance). **b** Graphical depiction of LysoTracker assay for assessing lysosomal membrane permeabilization. LysoTracker dye stains acidic compartments such as the lysosome. Depending on cell line, LMP damaged lysosomes can either be enlarged and have a higher fluorescent intensity or become damaged, leak, and/or dissolve which would be observed as lower fluorescence intensity using LysoTracker dyes. Using an LMP inducing agent (LLoMe or GPN) allows one to see the predicted dye retention of each cell line when a cell’s lysosomes are undergoing LMP. GPN was used for early experiments but due to the short shelf life, powdered LLoMe reconstituted fresh was later used. (All experiments in this figure were performed in triplicate and reported as geometric mean ± STD, Unpaired two-tailed *t*-test was used to determine statistical significance). **c** A graph representing the lysosomal health as measured by LysoTracker dye of FaDu WT and FANCD2-null cells expressing control non-targeting control (NTC-KD) or SNAP23 (SNAP23-KD) sgRNA in the presence or absence of LMP inducing agent (GPN). **d** A graph representing the lysosomal health as measured by LysoTracker dye of FaDu FANCA-null and FANCA-null rescued with overexpression of a WT FANCA transgene. LLoMe was used was used as LMP inducing agent. **e** The functional connections between FA pathway genes and lysosomal health were assayed in FaDu cells using LysoTracker. FANCA/B/F/L knockdowns are nonessential in FaDu and were assayed for effect in lysosomal and endosomal health for the FaDu cell line. FANCA/B/F/L knockdowns had statistically significant decreases in LysoTracker dye retention. **f** FITC-dextran was used to monitor early changes in lysosomal pH during the LMP process in FANCA/D2-null FaDu cells. The fluorescence intensity of FITC-dextran is reduced in normal lysosomes (pH 4–5) and upon LMP, neutralization of lysosomes leads to increase in fluorescence intensity. FITC-Dextran is overloaded into lysosomes then chased with fresh media and the fluorescence was measured using flow cytometry. LLoMe treatment was used as a positive control. **g** Lysosomal marker, LAMP-1 (green), was used to visualize lysosome number and morphology in FaDu WT and FANCD2-null with and without NTC or SNAP23 knockdown. Representative images are shown in (**g**) and quantification is shown in (**h**). **h** (Left) A plot of the distribution of the number of LAMP-1 foci per cell for FaDu WT and FANCD2-null with and without SNAP23-KD. (Right) A plot of the distribution of the average intensity of LAMP-1 foci for FaDu WT and FANCD2-null with and without SNAP23-KD. The open circles represent outliers defined by R base boxplot function as values > 1.5× outside the interquartile range (two-sided Mann–Whitney test was used to assess statistical significance).

Our findings suggest that deficiencies in the FA pathway result not only in the canonical DNA repair phenotype but can result in the reliance on genes involved in lysosomal exocytosis to maintain cellular homeostasis in certain cell types. Lysosomal exocytosis involves the secretion of lysosomal content by the fusion of the lysosome with plasma membrane. The fusion of the exocytotic lysosome into the membrane leaves a LAMP-1 scar on the cell surface. We used LAMP-1 luminal antibody and flow cytometry to quantify lysosomal exocytosis in FaDu WT and FANCD2-null cells with and without NTC and SNAP23 knockdown (Fig. 2a). FaDu FANCD2-null cells with NTC-KD showed ~1.5-fold increase in lysosomal membrane scars compared to WT background while both WT and FANCD2-null background with SNAP23 repression showed a decrease in overall lysosomal exocytosis. The FaDu FANCA-null cell line showed ~2-fold increase in surface LAMP-1 and the FANCA-null+Tg did not restore the lysosomal exocytosis rates to WT levels. Furthermore, lysosomal exocytosis of the FANCA/D2-null and FANCA-null +Tg in FaDu cells can be activated with ML-SA1, a chemical previously characterized to induce lysosomal exocytosis [18–20], which indicates we are studying the effects on lysosomal exocytosis (Supplementary Fig. S7).

In addition, we quantified lysosomal exocytosis with and without the loss of FANCA and FANCA-null + WT FANCA transgene rescue in UM-SCC-01. We found the LAMP-1 cell surface signal in the cell line with the FANCA-null to be increased compared to WT ~ 2.7-fold in UM-SCC-01 cells and is partially rescued with overexpression of FANCA transgene in FANCA-null background (Supplementary Fig. S8). The lack of rescue to partial rescue in FaDu and UM-SCC-01 FANCA-null with WT FANCA integrated cell lines could be due to non-endogenous expression levels of the FANCA transgene (Supplementary Fig. S9). Although the FaDu and UM-SCC-01 FANCA-null+Tg cell lines are an excellent resource provided by the FCF, the rescued cell lines have significantly higher levels of FANCA protein compared to WT, which can impact cellular phenotypes. The differences in expression are likely due to the use of non-endogenous promoters and the method of integrating a WT FANCA gene in a safe harbor location in the genome [21–23]. Overall, cells that have lost the FA pathway show increased rates of lysosomal exocytosis.

### Loss of the FA pathway exhibits defects in lysosomal health that result in lysosomal membrane permeabilization

#### Loss of FA pathway exhibits defects in lysosomal health

To maintain homeostasis, lysosomes break down and recycle or discard cellular proteins and products, and lysosomal exocytosis is one pathway for the cell to release unwanted material from lysosomes to the outside of the cell. Previous studies using zebrafish models of 3 different lysosomal storage disorders (Mucolipidosis II [MLII], sialidosis, and Mucopolysarchridosis Type IVA [MPSIVA]) showed that enhanced lysosomal exocytosis can arise from different genetic causes of lysosomal dysfunction [24]. We observed an increase in lysosomal exocytosis with the loss of the FA pathway and hypothesized lysosomal irregularities may be the cause for this observed phenotype. If lysosome dysfunction creates a reliance on the lysosomal exocytosis pathway to remove damaged lysosomes, the loss of the secretion pathway can cause lysosomes to become overwhelmed and/or break down and to release their contents into the cytosol through a process known as lysosomal membrane permeabilization (LMP) [9, 10] which can lead to cell death.

To functionally evaluate whether mutations in the FA pathway were altering lysosomal biology and potentially causing LMP, we used a previously established protocol to quantify lysosomal health and LMP using a lysometric dye, LysoTracker, that stains acidic compartments within a cell, such as lysosomes and endosomes [25] (See Methods). LysoTracker dye can be used to quantify the endosomal/lysosomal state of normal WT cells compared to mutant/altered cells. During LMP, protons leak through endosomal/lysosomal membranes which results in pH gradient changes that can be quantified using lysometric dyes such as LysoTracker. One potential result of LMP is lysosomes enlarge and will hold more lysometric dye and have a higher fluorescent intensity. Another result of LMP is lysosomes are damaged to the point of leaking protons and their inner contents which causes a pH gradient that can be captured as lower lysometric dye fluorescence intensity within the lysosome (Fig. 2b) [26–28]. Whether a cell line will have compromised lysosomes that swell or disintegrate is somewhat poorly understood and is cell line dependent, and thus an LMP inducing agent such as (GPN or LLoMe) is used as a positive control for such assays (Fig. 2b) [25, 26, 29]. The damaged lysosomes that undergo LMP release damaging lysosomal proteins and enzymes in the cell that cause cellular damage and eventually lead to cell death.

We observed that KD of SNAP23 in WT FaDu cells has a non-statistically significant effect on retention of lysometric dye relative to the non-targeting control KD and the LMP inducing agent caused lower retention of the dye (Fig. 2c). By contrast, FANCD2-null FaDu cells expressing a NTC sgRNA showed decreased lysometric dye retention in the lysosomes under baseline conditions relative to WT control cells suggesting an induction of LMP in the absence of FA-activity (Fig. 2c). Repression of SNAP23 in FANCD2-null FaDu cells further decreased lysometric dye retention suggesting dependency on SNAP23 for maintenance of lysosome health in the absence of FA proteins’ function.

This lysosomal defect is also seen with the FaDu and UM-SCC-01 FANCA-null cells (Fig. 2d, Supplementary Fig. S10). These defects could be partially rescued by WT FANCA transgene overexpression for FaDu cells and fully rescued in UM-SCC-01 cells (Fig. 2d, Supplementary Fig. S10). As mentioned before, the partial rescue observed in the transgene rescued cell lines could be due to non-endogenous expression levels of the FANCA transgene which can affect the observed biology (Supplementary Fig. S9). Likewise, supplementing with overexpression of SNAP23 did not rescue the phenotype in the FaDu FANCA/D2 deficient cell lines for LysoTracker dye retention (Supplementary Fig. S11).

We also tested the impact of loss of FA activity in different cell lines (FaDu, Cal33, UM-SCC-01, LN18, and LN229) on lysosomal health with and without the LMP inducing agent control (Supplementary Fig. S12). We observe a clear demarcation of the effect of LMP are cell line specific with LLoMe with the LysoTracker experiments: FaDu, Cal33, and LN18 with LMP inducing agent produce a phenotype of leaking/broken lysosomes while UM-SCC-01 and LN229 show a swelling of lysosomes. FaDu, Cal33, UM-SCC-01 are HPV negative head and neck cancer cell lines, while LN18 and LN229 are glioblastoma cell lines. Overall, the loss of FANCA in FaDu, Cal33, UM-SCC-01 and the repression of FANCD2 in LN18 had statistically significant defects in LysoTracker dye retention compared to WT conditions. Thus, we conclude knockout or knockdown of FANCA/D2 across diverse cellular models can induce lysosomal defect phenotypes.

#### The role of other FA proteins and DNA repair in lysosomal health

We then tested whether repression of other FA genes that are not essential for cell viability (FANCA/B/F/L) induced lysosomal and endosomal health phenotypes in FaDu cells. We found that repression of all FA genes tested had a statistically significant effect on lysosomal health relative to controls in FaDu cells (Fig. 2e). We also tested whether FA gene function is important for lysosomal health in a non-cancer cell line. For these experiments we used RPE-1 cells, which are a TERT-immortalized retinal pigment epithelial cell line [30] that is euploid and engineered to express CRISPRi. Here, we found that repression of FANCA and FANCD2 resulted in a significant elevated effect with the LysoTracker dye retention suggesting lysosomal health also depends on FA gene function in non-cancer cells (Supplementary Fig. S13).

To test if other DNA repair genes similar phenotypes, we knocked down four additional DNA repair associated genes (XRCC4, FBXO42, ATMIN, and BRD8) that have minimal effects on growth in the FaDu WT CRISPRi screen (depletion score > −1). We found that the repression of XRCC4, FBXO42, ATMIN, and BRD8 also resulted in statistically significant decreases in the LysoTracker dye retention compared to the NTC-KD which suggests altered lysosomal states (Supplementary Fig. S14).

Our results confirm previous reports and observations of general DNA damage causing cellular lysosomal/autophagy changes. Several studies have reported DNA damage triggers Golgi fragmentation [31, 32] and DNA-damaging anticancer drugs induce LMP and enhance cell death by damaged lysosome accumulation after addition of drugs that are autophagy inhibitors [31–35]. The emerging relationship between subcellular organelles and DNA damage response shed light on the complex regulatory networks that allow cells to respond to stress and maintain homeostasis.

#### Lysosomal membrane permeabilization is observed with the loss of FA pathway

To further explore the lysosomal defects and LMP, we employed various other assays to characterize LMP and the general lysosomal health of the loss of function FA mutants. LysoSensor was used to quantify the pH of the lysosomes and a LMP inducing agent (LLoMe) was used as a positive control to lysosomes that are undergoing LMP. The pH of the lysosomes in FANCA/D2-null are, as expected of lysosomes undergoing LMP, more basic compared to WT in FaDu and UM-SCC-01 cells (Supplementary Fig. S15a). The FANCA-null+Tg does not rescue the acidity in the UM-SCC-01 cell line but does in the FaDu cell line (Supplementary Fig. S15a). The proteolytic/lysosomal activity was measured using DQ-BSA Red and bafilomycin (an inhibitor of autophagosome-lysosome fusion) was used as a negative control. The proteolytic/lysosomal activity is higher in FA-null mutants in FaDu (FANCA/D2-null) and UM-SCC-01 (FANCA-null) cell lines. And the overexpression of a WT FANCA transgene in the UM-SCC-01 FANCA-null background did not rescue the proteolytic/lysosomal activity (Supplementary Fig. S15b).

To further characterize LMP in FA-mutant cells we used previously established protocols for pulse chase experiments with fluorescein isothiocynante-dextran (FITC-Dextran) [36, 37]. Fluorescein isothiocyanate (FITC) conjugated to dextran can be used to monitor early changes in lysosomal pH during the LMP process. The fluorescence intensity of FITC-dextran is reduced in normal lysosomes (pH 4-5). Upon LMP, neutralization of lysosomes leads to increase in fluorescence intensity. FITC-Dextran is first overloaded into lysosomes and chased with fresh media, which allows monitoring of changes in lysosomal pH during the LMP process. LLoMe treatment was used as a positive control to simulate the results of cells in the assay that are undergoing LMP. Cells undergoing LMP will show increases in fluorescent intensity as expected with LMP agent which can be measured by either flow cytometry or imaging [38]. The FA-null mutants showed statistically significant increase in FITC-Dextran fluorescent intensity in FaDu (FANCA/D2-null) and UM-SCC-01 (FANCA-null) compared to WT cells (Fig. 2f, Supplementary Fig. S16). The overexpression of a WT FANCA transgene in UM-SCC-01 FANCA-null shows a partial rescue of the phenotype using the FITC-Dextran assay.

Another signature characteristic of cells undergoing LMP is the translocation of Galectin-3 (LGALS3) from the cytosol to the lysosomal lumen [39–41]. Lysosome immunoprecipitation (Lyso-IP) was performed on the WT and mutant FaDu cell lines and the amount of LGALS3 was quantified using immunoblotting. An increase of LGALS3 was found in the lysosomes of the FANCA/D2-null mutants which corroborate the previous results (Supplementary Fig. S17). Another signature of damaged lysosomes undergoing LMP is the increase in K48-linked ubiquitination marks on lysosomes which signal engulfment by autophagosomes and recycling [42]. We found the lysosomes in the FaDu FANCA/D2-null cell lines had in increase in K48 ubiquitination (Supplementary Fig. S18). Overall, the data suggests that the defects in lysosomal health leads to LMP in cells deficient in the FA pathway.

From our data, we hypothesized that FA deficient cells may be more sensitive to chemical perturbations that cause lysosomal damage. There are well characterized chemicals that can cause lysosomal damage and LMP such as chloroquine (CQ). CQ preferentially accumulates in acidic organelles (lysosomes, endolysosomes, and Golgi), de-acidifies the luminal pH in acidic organelles, acidifies the cytosol and can cause LMP [43, 44]. Consistent with this hypothesis, we observed that FA-mutant cells are more sensitive to CQ than isogenic control cells in clonogenic cell survival colony formation assays. Specifically, we found that FANCA and FANCD2-null had increased sensitivity to CQ compared to WT in FaDu cells, 2.6 and 1.78-fold increase respectively (Supplementary Fig. S19). Cal33 FANCA-null similarly was more sensitive to CQ than WT cells (~1.76-fold sensitivity) and UM-SCC-01 FANCA-null had a ~1.26-fold change in sensitivity relative to WT cells that was not statistically significant.

### Loss of FA pathway causes differences in lysosomal morphology

To confirm the lysosomal defect, we used a lysosomal marker (LAMP-1- lysosomal associated membrane protein 1) to visualize lysosomal morphology in WT and FANCD2-null FaDu cells with and without NTC or SNAP23 knockdown (Fig. 2g, h; Supplementary Fig. S20 includes all data with LMP inducing treatment). Previous studies have shown that lysosomes and acidic organelles that undergo membrane permeabilization lose LAMP-1 puncta and staining fluorescence [45, 46]. In this assay, we stained and imaged LAMP-1 and then used NIS-Element GA3 software to compile the images into a Z-stack to identify LAMP-1 foci for quantification of foci count and fluorescence intensity (see Methods for details).

We observed a statistically significant decrease in LAMP-1 foci count in FANCD2-null+NTC-KD compared to WT + NTC-KD (Fig. 2h; ~−1.86-fold), corroborating that the lysosomes are undergoing LMP. The addition of a SNAP23-KD in FANCD2-null resulted in the lowest foci count compared to all other knockdown conditions (Fig. 2g, h). The deficiency in LAMP-1 foci in the FANCD2-null SNAP23-KD is likely due to the cell’s reliance on lysosomal exocytosis being blocked which results in the accumulation of lysosomal damage leading to LMP and eventual disintegration of the lysosome. In addition, the quantification of the mean intensity of the LAMP-1 foci per cell is significantly lower in the FANCD2-null vs. WT NTC-KD/WT SNAP23-KD. However, the SNAP23-KD in the FANCD2-null background shows a partial rescue in the mean LAMP-1 intensity per cell (Fig. 2g, h).

We hypothesize that FANCD2-null NTC-KD cells have lower LAMP-1 count due to defective lysosomes undergoing LMP, but these cells can survive due to the ability to secrete the damaged lysosomes. However, the loss of SNAP23 on top of the loss of FANCD2 results in the impairment of the cells’ ability to regulate lysosomal damage through exocytosis, which can lead to defective lysosomes being trapped and eventual lysosomal rupture and degradation [47]. Thus, the FANCD2-null SNAP23-KD results in decreased foci count and the semi-rescue of the LAMP-1 mean foci intensity.

### The loss of FA results in dysregulation of autophagy

FA proteins have a known role in mitophagy [48–51] and our findings of deficient lysosomes upon loss of FA proteins suggests a potential defect in general autophagy. Previous studies have shown that DNA damaging agents such as camptothecin [52], etoposide and temozolomide [53], p-Anilioaniline [54, 55], and ionizing radiation [55] arrest cell cycle and initiate autophagy. These findings show that even temporary activation of DNA damage can cause induction of autophagy. To explore potential autophagy deficiencies in the context of the FA pathway, we assessed mTORC1 activation by quantifying the levels phosphorylated 4E-BP1 (p4E-BP1) and found an increase in p4E-BP1 in FA mutants (FaDu FANCA/D2-null) compared to WT (Supplementary Fig. S21). The transgene rescued FANCA-null cell line (FANCA-null+Tg) also had increased levels of p4E-BP1 (Supplementary Fig. S21).

**Fig. 3:**
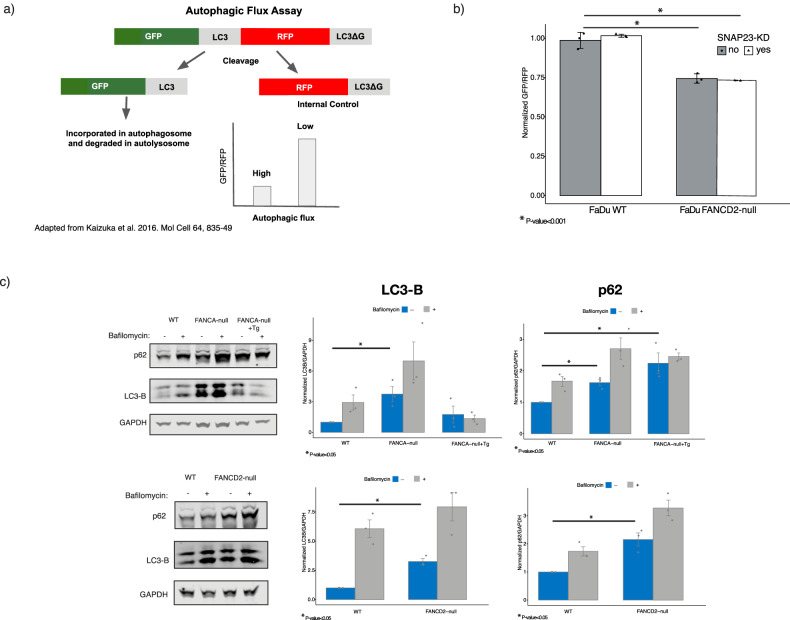
The loss of FA results in increased autophagic flux. **a** A schematic showing the GFP-LC3-RFP-LC3ΔG reporter assay used to assess the effect the loss of FANCD2 and FANCA on autophagic flux [57]. The autophagic flux is interpreted by comparing the mean fluorescence intensity of GFP/RFP. A lower value indicates high autophagic flux while a higher value indicates lower autophagic flux. **b** A graph quantifying autophagic flux in control cells and FANCD2-null cells with NTC or SNAP23 repression in the FaDu cell line (*p*-value < 0.001) (*n* = 3 as biological replicates; unpaired two-tailed *t*-test was used to determine statistical significance). **c** Levels of LC3-B and p62 were measured using western blot for FaDu FANCA/FANCD2-null and FANCA-null+Tg. Biological replicates were done for each set of conditions, and quantification of all replicates are plotted on the right (*n* = 3; unpaired two-tailed *t*-test was used to determine statistical significance).

The levels of p4E-BP1 indicated dysregulation of autophagy, thus leading us to analyze the rate of autophagic degradation (autophagic flux) [56]. We assessed autophagic flux upon loss of FANCD2 and FANCA activity using a fluorescent protein probe (GFP-LC3-RFP-LC3ΔG). Upon expression, this probe is cleaved by endogenous ATG4 family proteases resulting in equimolar GFP-LC3 and RFP-LC3ΔG that cannot undergo lipidation [57]. GFP-LC3 is degraded by autophagy and the RFP-LC3ΔG is released in the cytosol and serves as an internal control (Fig. 3a). To measure autophagy, the ratio of GFP/RFP fluorescence intensity is measured with lower values indicating higher autophagic flux while higher values indicate lower autophagic flux [57].

Using this assay, we tested autophagic flux in FaDu WT and FANCD2-null cells with and without SNAP23-KD. We found that FANCD2-null cells with and without SNAP23 repression exhibited a statistically significant increase in autophagic flux (Fig. 3b). Similarly, FANCA-null Cal33 cells also had increased autophagic flux compared to WT with and without SNAP23-KD (~1.2-fold increase; *p*-value > 0.005), Supplementary Fig. S22). These results show that the loss of FANCA or FANCD2 leads to increased autophagic flux that is not additive or synergistic with the loss of SNAP23. This may suggest that SNAP23 is not directly involved in the FA deficiency induced lysosomal defect.

To further characterize the autophagic flux changes in the FA mutants, we measured LC3-B and p62 levels. LC3-B is the insoluble, lipidated form of LC3 that is incorporated into the autophagosome while p62 is an autophagy receptor that binds to ubiquitin chains and signals targets to the autophagosome for degradation [56]. We found that the FaDu FA mutants (FANCA/D2-null) have accumulation of LC3-B and p62 (Fig. 3c). The overexpression of FANCA in the FANCA-null mutant in FaDu did not rescue the levels of LC3-B or p62 (Fig. 3c). This suggests that the FA mutants exhibit increased flux through impaired degradation of cargo.

### Lysosomal signature is clinically relevant in FA cancers

**Fig. 4:**
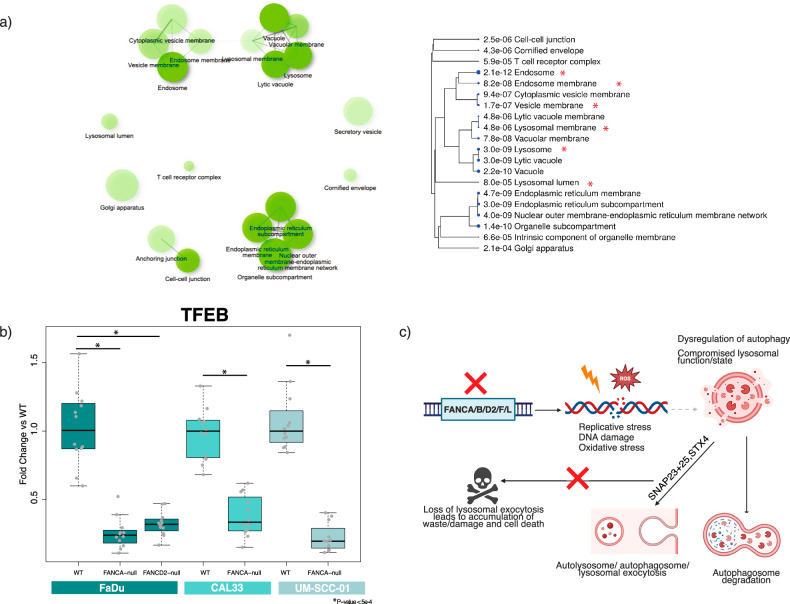
Transcriptomics of FA deficient patient samples demonstrate similar hallmarks of lysosomal and lysosomal exocytosis dysregulation. **a** Publicly available patient transcriptomic data that analyzed differential expressed genes from FA SCC patient tumors in comparison to sporadic HNSCC [58] confirmed a lysosomal/lysosomal exocytosis phenotype. All down regulated genes that had a statistically significant gene expression difference (*p*-value < 0.005) by at least a 1.5-fold were used as input for GO analysis (ShinyGO v0.77) [7]. The results show enrichment in lysosome related functions that are highlighted with red asterisks. **b** TFEB levels were measured using qPCR in FaDu, Cal33, and UM-SCC-01 FA-null mutants (*n* = 3 biological replicates each with *n* = 4 technical PCR replicates; Mean ± STD, unpaired two-tailed *t*-test was used to determine statistical significance). **c** A model of how deficiencies in FA pathway can lead to lysosomal defects and reliance on lysosomal exocytosis. The genomic stress that the loss of specific FA proteins causes replicative stress, DNA damage, and increase in oxidative stress. To combat these effects, there is dysregulation of autophagy and lysosomal biogenesis/health. This compromised state allows the cell to survive the loss of the FA pathway but renders the cell in a suboptimal state the is reliant on lysosomal exocytosis.

To determine if our findings in vitro are observed in clinical data from patients with FA disease, we used publicly available transcriptomic patient data to identify genes that are differentially expressed between FA squamous cell carcinoma (SCC) patient tumors in comparison to sporadic head and neck squamous cell carcinoma HNSCC tumors [58]. Ontology enrichment analysis on differentially expressed genes showed that genes that are upregulated in FA tumors predictably showed a statistically significant enrichment of DNA damage stimulus, DNA repair, DNA recombination, and DNA double-strand break (Supplementary Fig. S23). The analysis of the down regulated genes showed a statistically significant enrichment of genes that are involved with the lysosome and endosomal processes: lysosomal membrane (*p*-value < 4.8 e−6), lysosomal lumen (*p*-value < 8.0 e−5), endosome (*p*-value < 2.1 e−12), endosome membrane (*p*-value < 8.2 e−8), lysosome (*p*-value < 3.0 e−9), and vesicle membrane (*p*-value < 1.7e−7) (Fig. 4a).

The FA SCC clinical data similarly showed a statistically significant decrease in expression in key lysosomal and lysosomal exocytosis related genes: TFEB and mucolipin 1 (MCOLN1) in FA SCC patient samples compared to sporadic HNSCCs (−1.71 log2 fold change (*p*-value < 2.01 e −9) and −2 log2 fold change (*p*-value < 8.46 e−19), respectively) [58]. Notably, various lysosome specific proteins (LAMP-1/5) and enzymes (CTSB/D/F/G/S/W) were also found to be expressed significantly lower in FA SCC cancers (see Supplementary Table S8 [58]). TFEB is transcription factor that specifically targets the Coordinated Lysosomal Expression and Regulation motif (CLEAR) to regulate lysosomal biogenesis and function through the transcriptional control of 96 lysosomal genes [59]. By coordinating expression of lysosomal hydrolases, lysosomal membrane proteins, and autophagy proteins, TFEB can respond to lysosomal stress. In addition, TFEB is also known to play an important role in lysosomal exocytosis through Ca2+ signaling through MCOLN1. TFEB activates MCOLN1 to promote fusion of lysosomes to the plasma membrane to extrude lysosomal contents. TFEB is mainly localized in the cytoplasm and is translocated to the nucleus when activated [59]. The cellular localization of TFEB is controlled by phosphorylation status by mTORC1. mTORC1 phosphorylates TFEB leading to cytoplasmic localization. Starvation and lysosomal stress that leads to mTOR inhibition leads to TFEB nuclear translocation and activation of the lysosomal and autophagy genes [60, 61].

We confirmed the signatures we found in the FA SCC clinical data in our cell lines by measuring mRNA expression of TFEB and MCOLN1 in multiple in vitro FA-mutant and WT cell models. We observed substantial downregulation of TFEB (2.98-5-fold) across all FA-mutant cell models (FaDu, Cal33, UM-SCC-01) (Fig. 4b). A statistically significant decrease of MCOLN1 mRNA expression was also found in FANCA-null in FaDu and UM-SCC-01 (Supplementary Fig. S24a). We further validated our findings by quantifying TFEB and MCOLN1 protein levels in the FaDu cell lines. TFEB protein levels showed a statistically significant decrease while MCOLN1 protein levels did not in the FaDu FANCA/D2-null cell lines (Supplementary Fig. S24b). In addition, we quantified mRNA and protein expression of TFEB and MCOLN1 in patient derived immortalized FANCA/FANCD2-null fibroblast cell lines [62, 63] with and without a WT FA transgene rescue. The patient derived lines showed lower TFEB mRNA and protein levels but did not show the same trends in MCOLN1 compared to the transgene rescued cell lines (Supplementary Fig. S25a, b).

The localization of TFEB protein also plays a key role in the regulation of lysosomal biogenesis and autophagy. Phosphorylated TFEB is localized in the cytoplasm and upon dephosphorylation TFEB is translocated into the nucleus to induce transcription of the target genes. Thus, the ratio of cytoplasmic versus nuclear TFEB protein controls lysosome-autophagy pathway biology [64]. Imaging of TFEB in FANCA deficient FaDu and Cal33 cell lines showed statistically significant changes in nuclear/cytoplasmic TFEB intensity (Supplementary Fig. S26a, c). In addition, the immunofluorescence images confirmed lower total TFEB protein levels showing lower overall integrated TFEB fluorescence in both FA deficient FaDu (FANCA/D2-null) and Cal33 (FANCA-null) cell lines (Supplementary Fig. S26b, d). To determine if the lysosomal phenotypes were due to a reduction in TFEB levels, we transiently overexpressed TFEB and performed LysoTracker experiments. Consistent with the importance of proper TFEB localization, overexpression was not sufficient to rescue the lysosomal defect in FaDu FANCA/D2-null cells (Supplementary Fig. S26e). This suggests that loss of FA proteins impacts both levels and localization of TFEB with the latter being essential for proper lysosomal function.

Increased mTORC1 activity and decreased TFEB expression/nuclear localization are both associated with reductions in autophagy, which has been seen during persistent starvation leading to negative feedback on autophagy [65]. TFEB and mTORC1 also have a complex relationship with TFEB as a target for mTORC1 and mTORC1 localization to lysosomes being impacted by TFEB-dependent processes such as endocytosis [66, 67]. We suspect that the stress of the losing FA pathway causes similar activation and dysregulation of mTORC1 and TFEB. Overall, the FA patient gene expression data support our experimental findings that loss of FA pathway can cause defects in lysosomes and dysregulation of autophagy.

## Discussion

We set out to better understand the biological implications of FA pathway loss to identify potential therapeutic targets for HNSCC malignancies associated with FA that do not involve the use of DNA damaging agents. Our findings reveal that deficiencies in the FA pathway can lead to significant autophagy and lysosomal defects. The phenotype that we have characterized may be a consequence of the inherent DNA damage deficiencies and replicative stress in FA deficient cells.

The crosstalk between DNA damage and autophagy has been reported to contribute to or prevent cell death depending on context [68, 69]. To further add to the complexity, autophagy is a major degradation system that derives its regulatory abilities through lysosomes and has a role in dysfunctional mitochondrial regulation (mitophagy). Autophagy can mitigate and protect against DNA damage by regulating the recycling of the DNA repair proteins, mitochondrial quality control, controlling ROS production, ATP production, and cell death signaling [60].

The complex connection between ROS and the autophagy-lysosomal system is well-documented but the mechanism behind these interactions remains elusive [70–74]. The impairment of lysosomes is closely intertwined with excessive accumulation of ROS and this can impact lysosomal acidity and modulate autophagic processes. Proton pumps such as H+-ATPase serve as a way of maintaining lysosomal acidity and ROS can stimulate the activity of proton pumps and accelerate the cycling of H+ ions and increasing lysosomal acidity [75, 76]. Other acid pumps such as Na+/H+ exchange channels and Cl−/H+ exchange channels can be impacted by ROS accumulation and similarly disrupt lysosomal acidity. Previous studies have shown that the increased ROS levels can regulate autophagy through several autophagy related pathways. The pathways include the activation of AMPK signaling cascade and the ULK1 complex, oxidation of Atg4, disruption of the Bcl-2/Beclin-1 interaction, and alteration of mitochondrial homeostasis and membrane depolarization leading to mitophagy [77]. ROS can also regulate autophagy indirectly through dysregulation of mTORC1 [78].

FA proteins are primarily known to be involved in DNA repair and thought to cause diseases due to deficiencies in DNA repair and replicative stress which can also indirectly result in increased reactive oxygen species (ROS) [70, 79]. The complex factors of DNA damage, ROS, and the network of interactions through autophagy and lysosomal/mitochondrial states may explain the defects that we have identified and characterized in our study. We confirmed the DNA damage phenotype seen in FA deficiency by testing by quantifying γH2AX. The FA-null mutants for FaDu (FANCA/D2) had an increase in basal γH2AX (Supplementary Figs. S3, S27). The FANCA-null+Tg cell lines also showed an increase in γH2AX despite the overexpression of the FANCA transgene. The inability of the transgene to rescue γH2AX levels may explain the partial to no rescue we see in our study. We confirmed in our cell lines that FANCA and/or FANCD2- null cell lines (FaDu, Cal33, and UM-SCC-01) have increased ROS levels (Supplementary Fig. S28) and increased mitochondrial ROS (mtROS) (Supplementary Fig. S29a). In addition, we used a mitochondrial membrane potential dye (JC-10) to assay mitochondrial defects in FaDu FA-deficient cells. We found FaDu FANCA/D2-null cell lines have defects in mitochondrial membrane potential (Supplementary Fig. S29b).

Altogether, the genotoxic stress and increase in ROS with the consistent loss of DNA repair activity may indirectly necessitate dysregulation of lysosomal state and autophagy to compensate and reach homeostasis to sustain viability. This compromised autophagic and lysosomal state may be dependent on controlling lysosomal biogenesis and autophagic state that relies on dealing with damage by using lysosomal exocytosis which results in dependency of SNAP23/STX4 for survival (see model: Fig. 4c).

While we do not explore how defects in the FA pathway ultimately lead to these lysosomal phenotypes and dysregulated autophagy, it is unlikely that the connections are direct. Crosstalk between exogenous chemical and DNA damaging agents (camptothecin [52], etoposide & temozolomide [53], ionizing radiation [54]) have been shown to dysregulate autophagy. These studies reported the autophagic changes that result from induced DNA damage response are temporary. In contrast, the total loss of the FA pathway leaves cells less able to cope with endogenous forms of DNA damage (e.g. ICLs from aldehydes [80]) and to protect replication forks [81–83]. These cells are likely in a somewhat persistent state of stress, and we speculate that the loss of FA pathway forces cells to alter the dynamics of autophagic processes and lysosomal quality control to cope, which leads to a dependency on lysosomal exocytosis. Overall, our findings contribute to a broader understanding of the FA pathway and allows us to expand our understanding of how mutations in FA genes contribute to the pathology of the disease in the context of lysosomal health and lysosomal exocytosis.

## Methods

### Cell lines

Many of the cell lines used in this study were purchased from American Type Culture Collection (ATCC): FaDu, LN18, LN229, and RPE-1. FaDu were grown in RMPI 1640 (Gibco). LN18, LN229, and RPE-1 were grown in DMEM. All media was supplemented with 10% fetal bovine serum (FBS) with 1X penicillin-streptomycin-glutamine(pen-strep-glut) [Thermofisher #10378016] and grown in a humidified 5% CO_2_ incubator at 37 °C. See Supplementary Table S10 for a list of cell lines.

FaDu FANCD2-null was made by electroporating Cas9 ribonucleoproteins (RNPs) into WT FaDu dCas9-KRAB cell line using the Lonza Amaxa (ATCC HeLa presets). Candidate cell lines were isolated and established as clonal cell lines. Each candidate cell line was checked for the loss of function FANCD2 protein via immunoblotting.

FaDu and UM-SCC-01 cell lines WT, FANCA-null, and FANCA-null+ WT FANCA transgene were generously donated by the FCF (www.fanconi.org; Fanconi Cancer Foundation). In addition, Cal33 WT and FANCA-null was also donated from FCF. UM-SCC-01 and Cal33 were grown in DMEM + 10% FBS + 1X pen-strep-glut.

### CRISPRi cell lines

CRISPRi cell lines were made according to previously published protocols [84, 85]. Briefly, WT and FANCD2-null cell lines were infected with virus made from dCas9-KRAB construct (Addgene #217304) at low MOI (~30%). Each cell line was sorted for dCas9-KRAB using the BFP marker in the construct and validated for activity using an essential compared to NTC sgRNA in a depletion assay (see Validation/competition assay for details).

### Lentivirus production

All lentivirus made in this manuscript follows the Weissman lab protocol “Large scale lentivirus production protocol” (weissman.wi.mit.edu/crispr/). In summary the gag & pol (dR8.91), env (MD2G), lentiviral plasmid was transfected at an 8:1:8 μg ratio for 15 cm plate of 293T cells. The ratios were modified proportional to plate surface area for 10 cm plates.

### CRISPR screening and data analysis

Whole genome screens using the open source CRISPRi V2 library (Addgene #1000000091) were performed in FaDu WT and FANCD2-null background and published screening protocols were followed for CRISPRi screens [85, 86]. The genome scale library virus was generated and transduced at low MOI such that each cell expresses on average one sgRNA and thus one gene is knocked down per cell (~30% infected). After 72 hrs of infection, cells were selected on 5 μg/ml Puromycin. The sgRNA containing cells were expanded to establish 2- T0 and 2- T-final replicates each at 500× coverage. The 2 replicates were passaged for 14 days (~10 population doublings) to allow for knockdown of genes to manifest as growth phenotypes in the presence or absence of FANCD2. Each replicate cell line was maintained at 500× coverage and 500× coverage was frozen down for the T-final. The samples were prepped using the Macherey Nagel NucleoSpin Blood kit and protocols. The quantity of each sgRNA in each sample (T0, T-final in WT and FANCD2-null) was quantified by sequencing and then sgRNA and gene level phenotypes were calculated for each screen.

The depletion scores were calculated using Weissman lab ScreenProcessing protocol [85]. The collapsed gene depletion scores, raw counts and phenotype table can be found in for FANCD2-null can be found in Supplementary Tables S4–6 and for WT, Supplementary Tables S7–9. To compare depletion scores between WT and FANCD-null, depletion scores were quantile normalized using the R package preprocessCore [87]. Quantile normalization was implemented to minimize technical variability. As stated above, FANCD2-null specific candidates of interest were filtered to have depletion score lower than −1.5 and *p*-value < 0.005 in the FANCD2 screen and no growth effect in the WT background.

### Validation/competition assays

To validate candidates, individual sgRNAs were cloned into the sgRNA vector for each gene of interest. The sgRNA vector used was either the BFP or mCherry sgRNA derived from pU6-sgRNA EF1Alpha-puro-T2A-BFP (Addgene #60955) [85]. Each sgRNA candidate was cloned using BlpI and BstXI sites (detailed protocol found in https://weissman.wi.mit.edu/crispr/). WT and FANCD2-null cell lines were infected with the virus from each sgRNA candidate at a MOI ranging from 30 to 50%. The percentage of infected cells for each were tracked over 10–14 doublings using either a BFP or mCherry marker on the sgRNA plasmid using a flow cytometer (Attune NxT). The competition assay was performed using one sgRNA harboring the BFP and the other candidate with mCherry. WT and FANCD2-null cell lines were infected with each individual sgRNA and both. The percentages of each individual sgRNA and both were tracked via flow analysis. Depletion scores were calculated by comparing log2 of the initial infection percentage and subsequent time points and end time point (see Fig. 1a). Similarly, for validations the sgRNA candidate was tested against a NTC control, in this case a GAL4 sequence (5’-TTGGACGACTAGTTAGGCGTGTA-3’).

### Autophagic flux GFP-LC3-RFP-LC3ΔG assay

The GFP-LC3-RFP-LC3ΔG assay was established in the CRISPRi FaDu (WT & FANCD2-null) and Cal33 (WT & FANCA-null) using a modified pMRX-IP-GFP-LC3-RFP-LC3ΔG (Addgene #84572) [57]. The GFP-LC3-RFP-LC3ΔG was cloned into a lentiviral vector (pFU-227). In short, lentivirus was made from the construct and infected at a low MOI in each cell line. Each infected cell line was then sorted for GFP and RFP and expanded. FaDu and Cal33 cell lines were then each infected with either NTC or SNAP23 sgRNA. After infection, GFP and RFP levels were measured via flow cytometer (Attune NxT).

### Lyso-IP experiments

Cell lines were made according to previously published lysosome immunoprecipitation (Lyso-IP) protocol [88]. Cell lines were infected with pLJC5-Tmem192-3xHA (Addgene #102930) to introduce the lysosomal tag and was selected on Blasticidin (10 μg/ml). After cell lines are established, cell lines were expanded to 30 million cells for each cell line (FaDu-WT, FANCA-null, FANCD2-null) with and without 1 mM LLoMe treatment (30 min). Cells are rinsed twice with PBS and scraped with KPBS (136 mM KCl, 10 mM KH2PO4, pH 7.25 was adjusted with KOH). Cells were homogenized with a dounce homogenizer. The homogenate was centrifuged at 1000 × *g* for 2 min at 4 °C. The supernatant was removed and incubated with 50 μl of anti-HA magnetic beads (pre-washed with KPBS). The lysates were quantified using BCA assay (Thermofisher #23227) and 2000 μg lysate and HA beads were incubated at room temp on a rotator for 15 min. After incubation, the beads were washed 3 times with KPBS and resuspended in 50 μl of Laemmli buffer and used for western blotting (Galectin-3 [BD Biosciences 556904], LAMP-1[Cell Signaling D5C2P]).

### Western blotting

Protein from cells were lysed in RIPA buffer (Milipore 20188) with phosphatase and protease inhibitor (Thermofisher scientific #PI788445). Lysates were run on Bolt Bis-Tris Mini Protein gels (4–12%) on 0.45 μm nitrocellulose membrane. Each experiment was performed at least three times, and representative images are shown (see Supplementary Table S11 for list of antibodies).

### qPCR

At least 1 million cells were plated at ~50–60% confluency overnight and harvested the next day. Cells were trypsinized and washed 2× with PBS. The RNA was extracted from each sample with the Macherey-Nagel RNA isolation kit (#740955.50). RNA was quantified and (~100 ng) reverse transcribed using Thermo Scientific RevertAid cDNA synthesis kit (#K1621). For the reverse transcription 1 cycle of 25 °C (10 min), 37 °C (60 min), and 95 °C (5 min) was followed. After reverse transcription, qPCR was done using Taqman Fast Advanced Master Mix (#4444963). The qPCR experiment and analysis were done using the Roche LightCycler 480-II. All primers were ordered from IDT Predesigned PrimeTime™ qPCR primers.

### LysoTracker/LysoSensor flow assays

Cells were counted using the Attune NxT and plated in a 12 well and allowed to adhere overnight. All FaDu derived lines were plated at 20 K due to the cell lines apparent preference for higher density. While all cell lines derived from Cal33, LN18, LN229, UM-SCC-01, and RPE-1 were plated at 10 K. Enough cells were plated for triplicates for each cell line plus and minus LMP induced treatment. For a positive control arms, original media is removed and replaced with 1 ml of 333 nM LLoMe (Cayman chemicals) or GPN (MedChemExpress) were added for 15–20 min. GPN was used for early experiments but due to the short shelf life, powdered LLoMe reconstituted fresh was later used. After LMP inducing treatment, 50 nm LysoTracker Red (Thermofisher #L7528) or Lysosensor Green (Thermofisher #L7535) dye in each well for 30 min–1 hr. After incubation, cells were washed 2× times with PBS and harvested using trypsin and resuspend in PBS + 1% Fetal Bovine Serum. Samples were analyzed using the Attune NxT, using the YL2 channel for LysoTracker red and BL1 channel for LysoSensor. The geometric mean was calculated for all samples, and a median was taken.

### Colony formation assays

Each cell line of interest was counted using the Attune NxT and 10 K cells were seeded into each well of a 6 well. The cells were allowed to adhere overnight, and the next day varying doses of CQ resuspended in DMSO and a DMSO control was added. Fresh CQ was replace every 3^rd^ day for 3 times (9 days total). When assay in finished, cells were washed twice with PBS and 500 μl of 0.5% crystal violet fixation solution (0.5 g crystal violet, 20 ml of methanol, 80 ml of water) was added in each well for 15–30 min. Colonies could be visualized after washing with water 3–5× times.

### DQ-BSA pulse chase flow assay

Cell lines were counted and plated at 20 K in a 24-well plate for treated and untreated conditions and allowed to adhere overnight. For a negative control arms, original media is removed and replaced with 10 μM Bafilomycin (MCE #HY-1000558) and incubated for 2 hrs. After 2 hrs of Bafilomycin treatment, cells were incubated with a master mix of DQ-BSA Red (Thermofisher #D120451) for 1 hr. After 1 hr of DQ-BSA (1 μg/ml) chase, the media was changed and allowed to chase for 3 hrs. When chase is finished, cells were washed with PBS 2× and then lifted for flow analysis.

### Dextran-FITC pulse chase flow assay

Cell lines were counted and plated at 20 K in a 24-well plate and allowed to adhere overnight. After overnight incubation, old media was removed and either new media with Dextran-FITC or Dextran-FITC + 1 mM LLoMe (positive control) was added for a 2 hr pulse [Dextran-FITC (AAT Bioquest #21700) 5000 μg/ml]. After the 2 hr incubation the media was removed, and fresh media was added for an overnight incubation. The next day at 24 hrs, the cells were washed with PBS 2× and lifted for flow analysis.

### Caspase 3/7 flow assay

CellMeter™ Caspase-3/7 Green Flow (AAT Bioquest #22823; optimized for flow cytometry) to quantify the activity of caspase 3 and 7. We used 10 μM Mitomycin C (MMC), a chemical known for inducing apotosis [89], overnight treatment as a positive control. Per manufacturer recommendation, 500X TF2-DEVD-FMK solution was diluted to 1X Assay buffer. Cells were incubated with 1× working buffer Caspase-3/7 dye for 1 hr. After Caspase-3/7 incubation, cells were washed with PBS and lifted for flow analysis.

### JC-10 flow assay

CellMeter™ JC-10 Mitochondrion Membrane Potential Assay Kit (AAT Bioquest #22801; optimized for flow cytometry) was used to quantify mitochondrial membrane potential. Cells were treated with rotenone as a positive control (4 hrs, 4 μM). JC-10 is based on the detection of mitochondrial membrane potential changes in cells by the cationic, lipophilic JC-10 dye. JC-10 concentrates in the mitochondrial matrix where it forms red fluorescent aggregates. Per manufacturers recommendation, a working solution was made by adding 25 μl of 200X J-10 into 5 mls of Buffer A. Cells were incubated in 500 μl of JC-10 working solution for 1 hr. Finally, cells were washed with PBS and lifted for flow analysis.

### TUNEL flow assay

DNA fragmentation was quantified using a TUNEL assay (AAT Bioquest #22849). Per manufacturers recommendation, a master mix of 0.5 μl of 100X Tunnelyte was added int 50 μl of Reaction buffer to make 50.5 μl of TUNEL working solution for each sample. Cells were incubated with 50 μl of TUNEL working solution for 1 hr. After 1 hr, the working solution was removed, and cells were washed with PBS and lifted for flow analysis.

### ROS assays

The Attune NxT was used to count cells to plate in 12 well plate. All FaDu derived lines were plated at 8 K due to the cell lines apparent preference for higher density. While cell lines derived from Cal33 and UM-SCC-01 were plated at 5 K. Enough cells were plated for cell line for triplicates plus and minus positive control. For the ROS assay CellROX Green (ThermoFisher #C10444) was used and for the MitoSOX Red (ThermoFisher #M36007) was used. Positive controls for CellROX were treated with hydrogen peroxide for 10 min (1:1000 dilution of 30% peroxide in media). Positive controls for MitoSOX were treated with 50 μM mitoparaquat overnight. For the experiment, 500 nM CellROX or MitoSOX was incubated with the cell lines for 1 hr. After incubation, cells were wash with PBS 2× and lifted and resuspended with PBS + 1% Fetal Bovine Serum cells for flow cytometry.

### Surface LAMP-1flow assay

About 100k cells were fixed with 4% paraformaldehyde for 15–30 min and washed twice with PBS. The cells are resuspended in PBS + 10% fetal bovine serum (FBS) with no antibody (control) or with Santa Cruz Biotechnology anti-rat LAMP1-(H4A3 1:100; sc-20011) at 4 °C for at least 60 min. All cells were then washed PBS and incubated with Alexa-647 conjugated anti-rat secondary for at least 30 min at room temperature in PBS + 10% FBS (1:1000) protected from light. Cells were then washed with PBS twice and resuspended in PBS + 10% FBS for flow cytometry analysis (Attune NxT). The mean fluorescence of each condition was measured. To normalize for background fluorescence, each condition with the antibody+secondary was subtracted by the median of mean fluorescence of the corresponding condition’s control with only secondary. For plotting, all conditions were normalized by dividing by the median of the WT condition’s mean fluorescence. For ML-SA1 activation, cells were incubated with 50 mM ML-SA1 and processed as previously described.

### Immunofluorescence microscopy and analysis

#### Immunofluorescence microscopy

Cells 150 K cells for each cell line was plated on poly-L-Lysine coated cover slips and allowed to adhere overnight. For positive controls hydrogen peroxide (1:1000 dilution of 30% peroxide in media) was incubated with each slide for 10 min. All coverslips were fixed with 4% paraformaldehyde at room temperature (RT) for 15–30 min. Coverslips were then treated with blocking buffer (1.1% BSA, 0.1% Triton X-100 in PBS) for 15–30 min at RT. Each coverslip was incubated overnight in 4 °C in 100 μl of 1:200 of LAMP-1 antibody (Cell Signaling Technology D4O1S anti-mouse). After primary antibody incubation, coverslips were washed 3× 5 min with PBS + 0.1% Triton-100. Secondary antibody was added at a 1:500 dilution + DAPI for 1 hr at RT. Finally, coverslips are washed 2 × 5 min with PBS + 0.1% Triton-100 and 2 × 5 min with PBS. Coverslips were sealed with Invitrogen ProLong Gold AntiFade and allowed to dry and were kept in –20 °C and away from light. Images and data for this study was acquired at the Center for Advanced Light Microscopy- Nikon Imaging Center at UCSF on Spinning Disk confocal microscope.

#### Analysis

IF analysis was performed using GA3 in NIS-Element. Foci segmentation was done with NIS.ai in NIS-Element. Whole cell segmentation was done by applying ‘Grow Regions’ module in GA3 from nucleus area (segmented from maximum projected DAPI images by Otsu thresholding) to whole cell area. The count, volume, and intensity of LAMP-1 foci were assessed.

## Summary

Using unbiased CRISPRi screening, we found the loss of Fanconi anemia (FA) pathway proteins leads to deficiencies of lysosomal biology and autophagy. These defects are marked by dysregulation of TFEB, lysosomal membrane permeabilization, and a dependency on lysosomal exocytosis. Our findings are the first to link the FA pathway to major changes in lysosomal and autophagic state to a dependency on lysosomal exocytosis. Our findings expand our understanding of FA as a disease and of induced dependencies in FA mutant cancers.

## Supplementary information

Supplemental Material

Table S1

Table S2

Table S3

Table S4

Table S5

Table S6

Table S7

Table S8

Table S9

Table S10

Table S11

Original data

## Data Availability

The unprocessed screen data has been deposited in the public database Sequence Read Archive (SRA accession: PRJNA1305101).
