# Supplementary material for: Loss of Fanconi anemia proteins causes a reliance on lysosomal exocytosis
Source: Cell Death Dis. 2025 Nov 4;16(1):791. doi: 10.1038/s41419-025-08164-0 (PMC12586712; doi:10.1038/s41419-025-08164-0)
Supplement: Supplementary file 13 — Original data [file 41419_2025_8164_MOESM13_ESM.pdf]

Supplementary Western blots:

Figure 3)

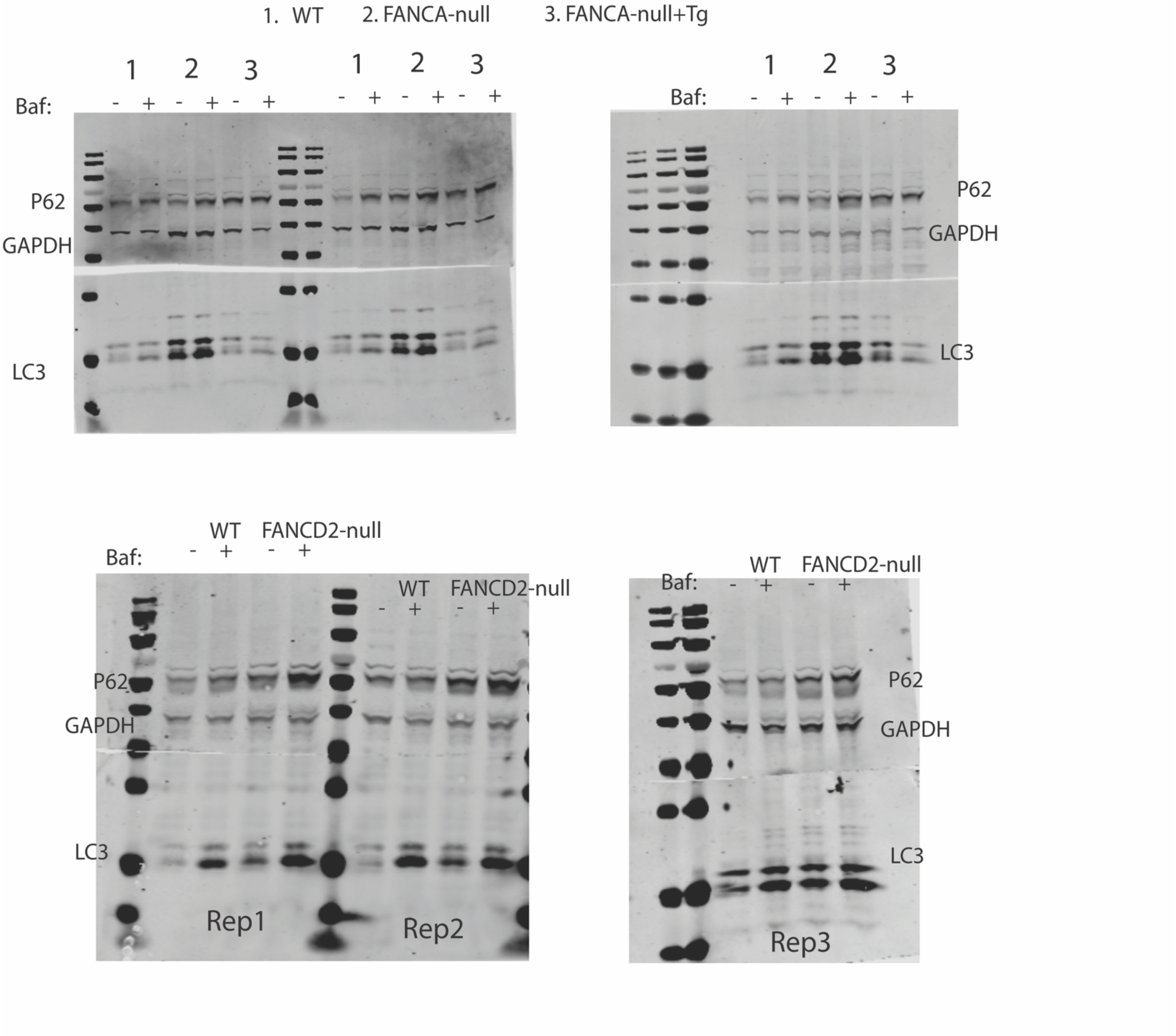

Figure S1)

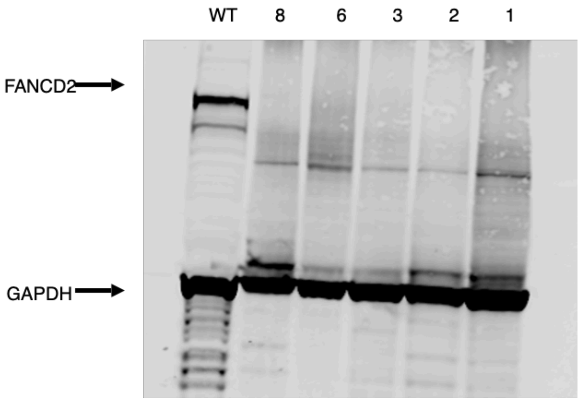

Figure S3)

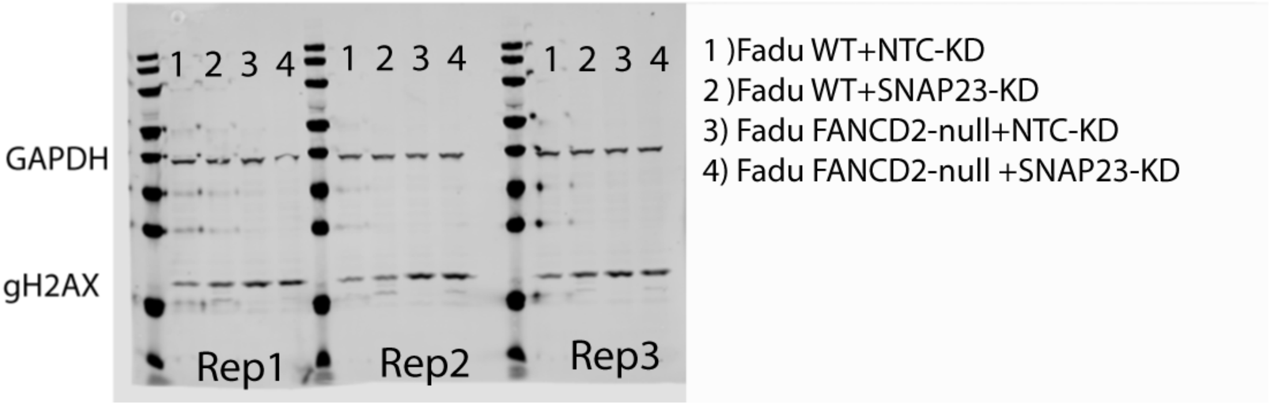

Figure S9)

FaDu:

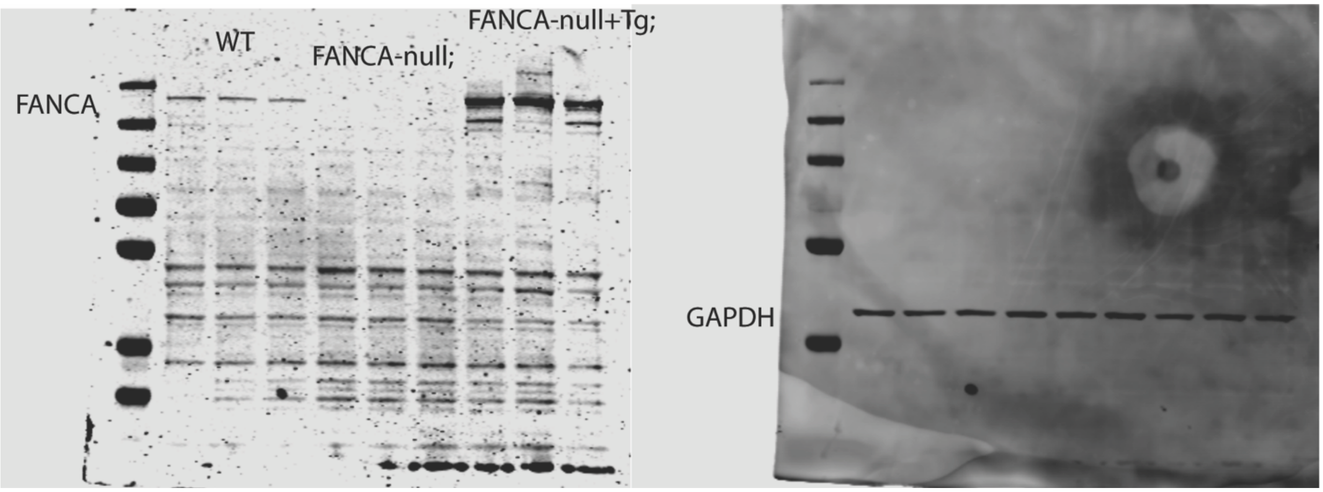

UM-SCC-01:

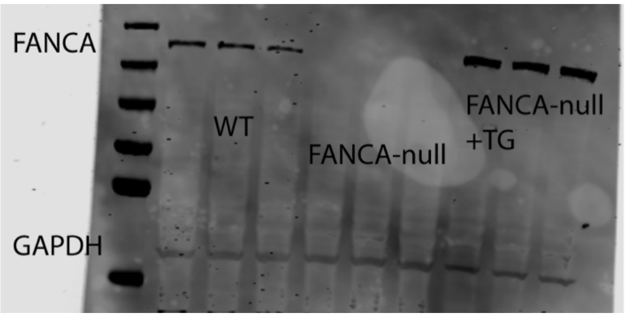

Figure S17)

FANCA-null LysoIP:

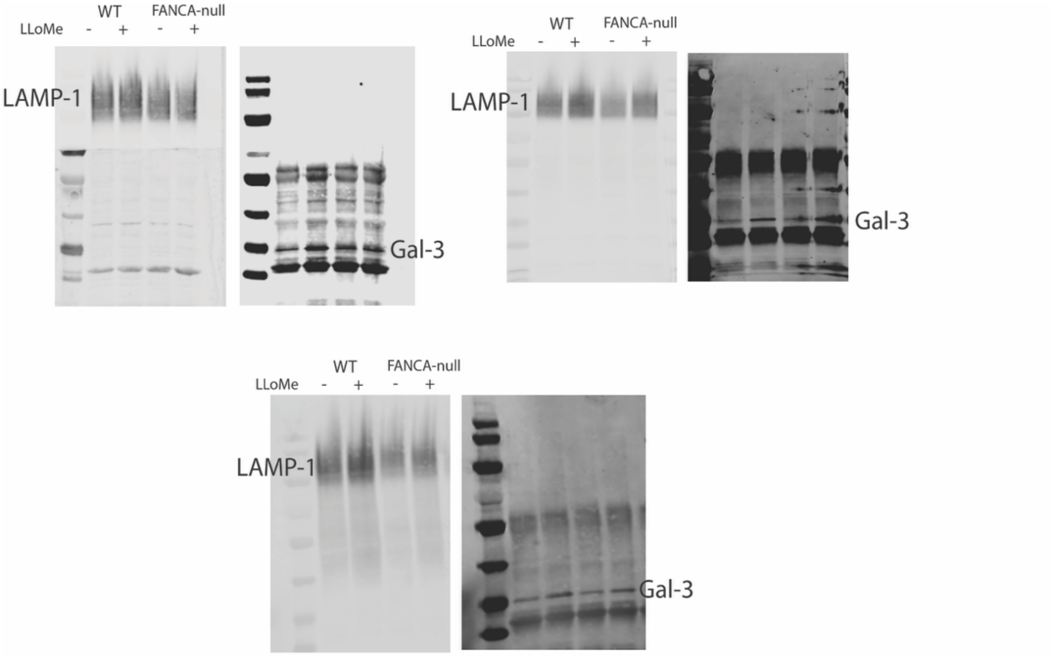

FANCD2-null LysoIP:

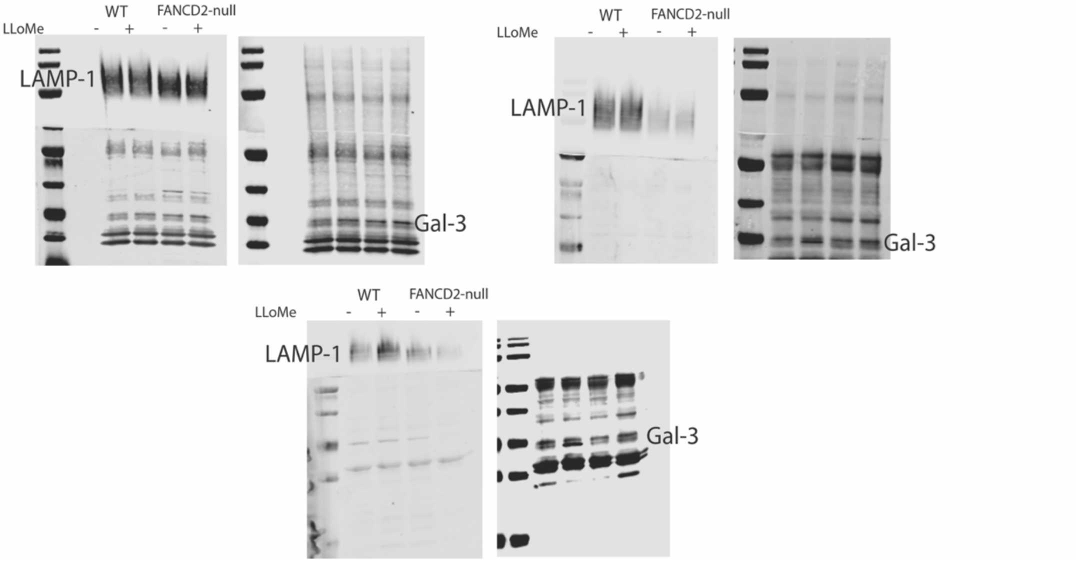

Figure S21)

FANCA-null:

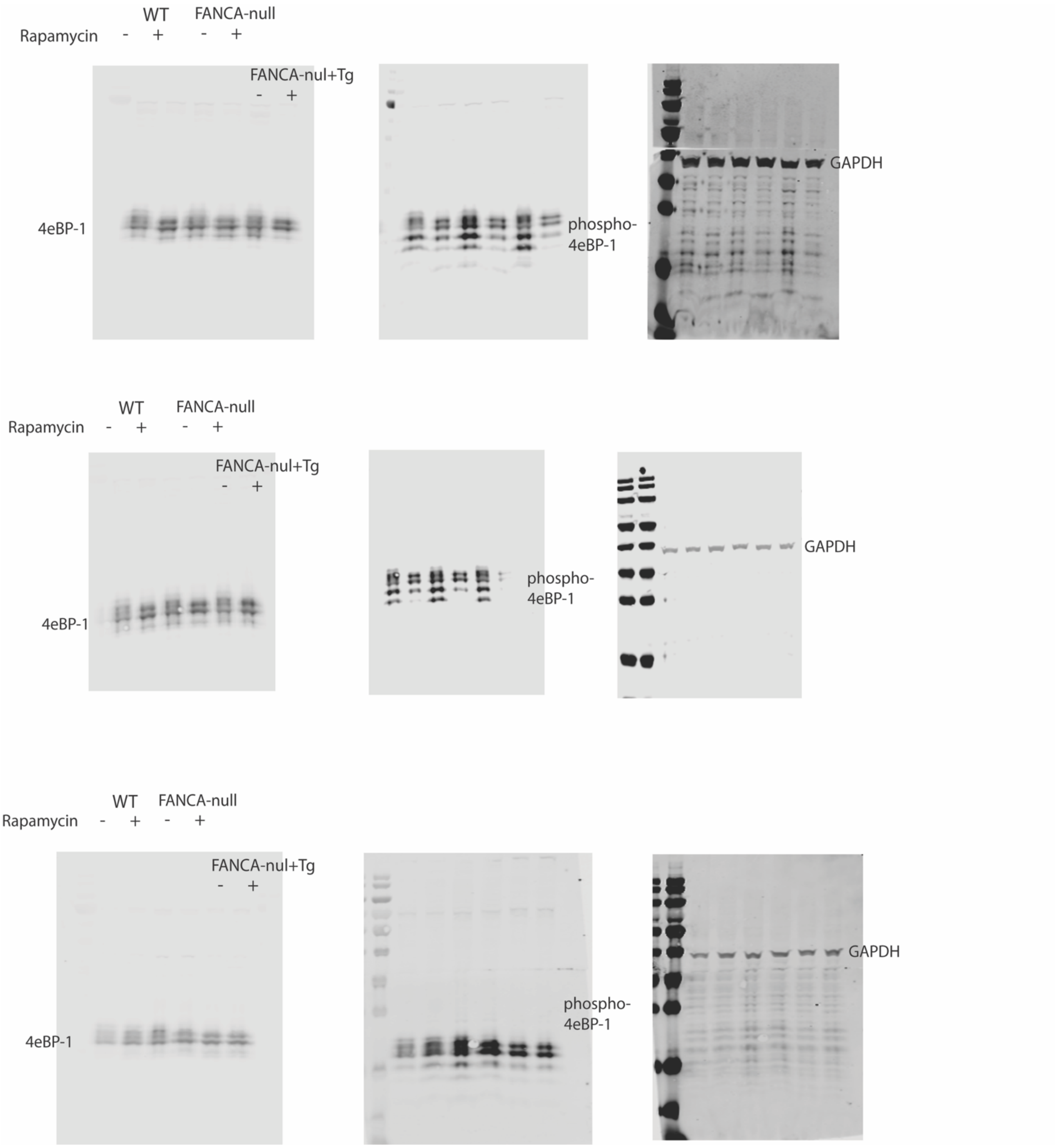

FANCD2-null:

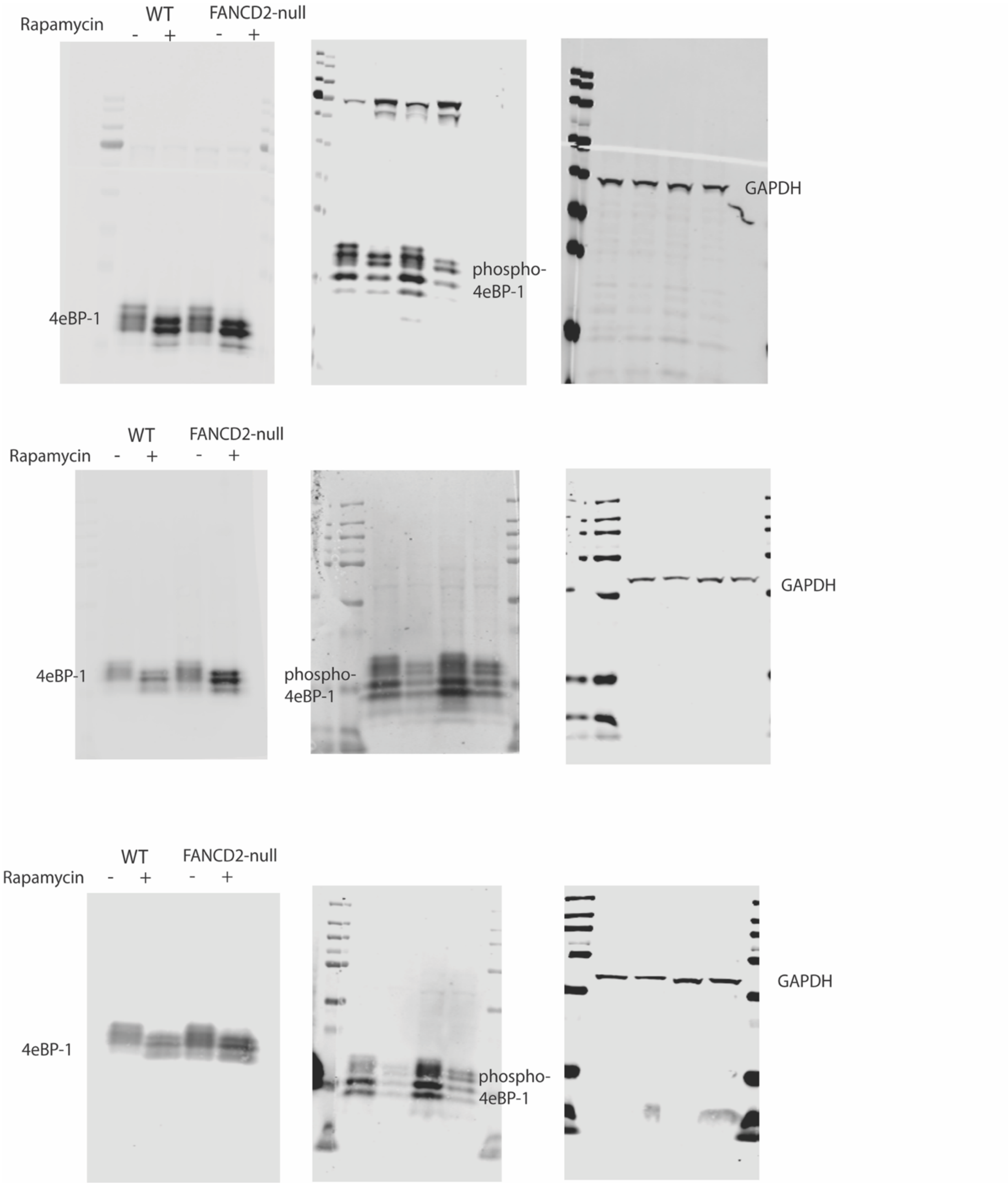
WT
FANCD2-null
Rapamycin
- + - +
4eBP-1
phospho-
4eBP-1
GAPDH
WT
FANCD2-null
Rapamycin
- + - +
4eBP-1
phospho-
4eBP-1
GAPDH
WT
FANCD2-null
Rapamycin
- + - +
4eBP-1
phospho-
4eBP-1
GAPDH

Figure S24b)

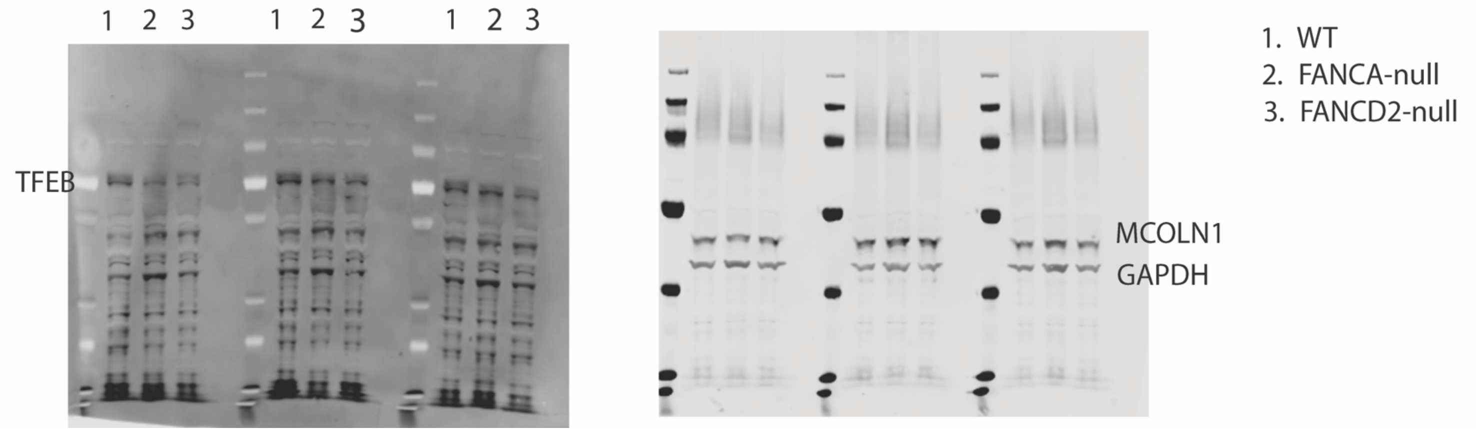

Figure S25)

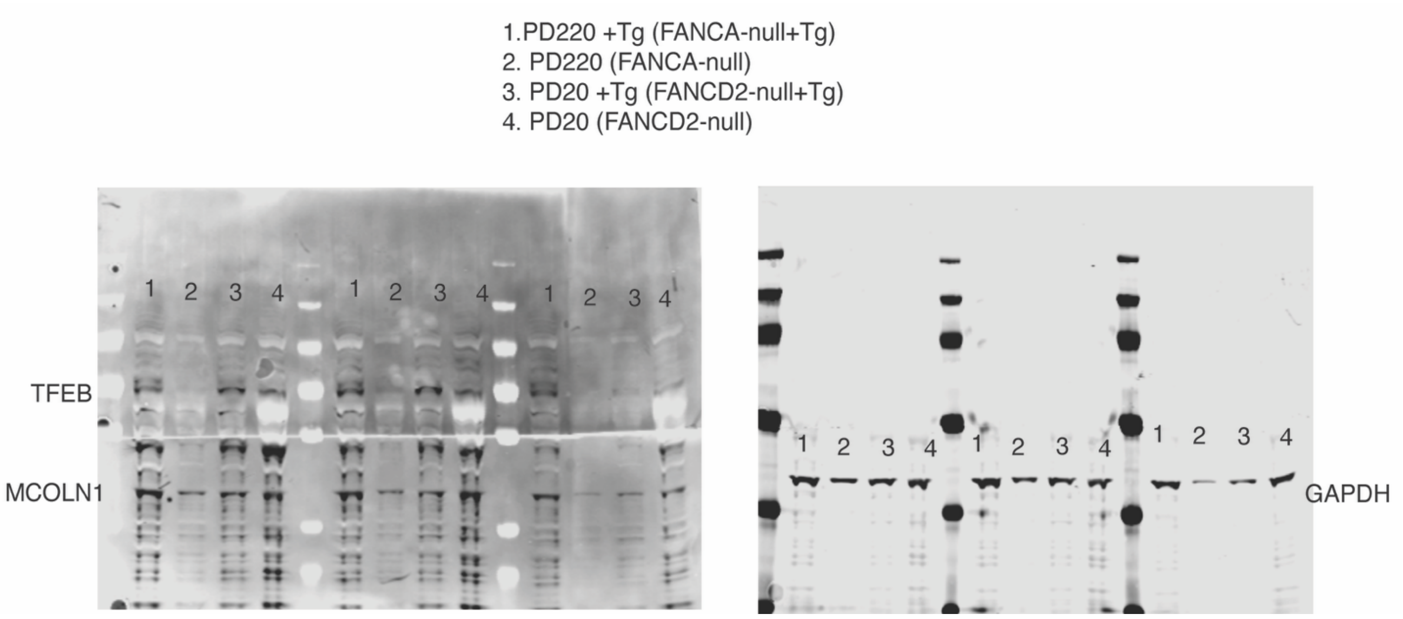

Figure S27)

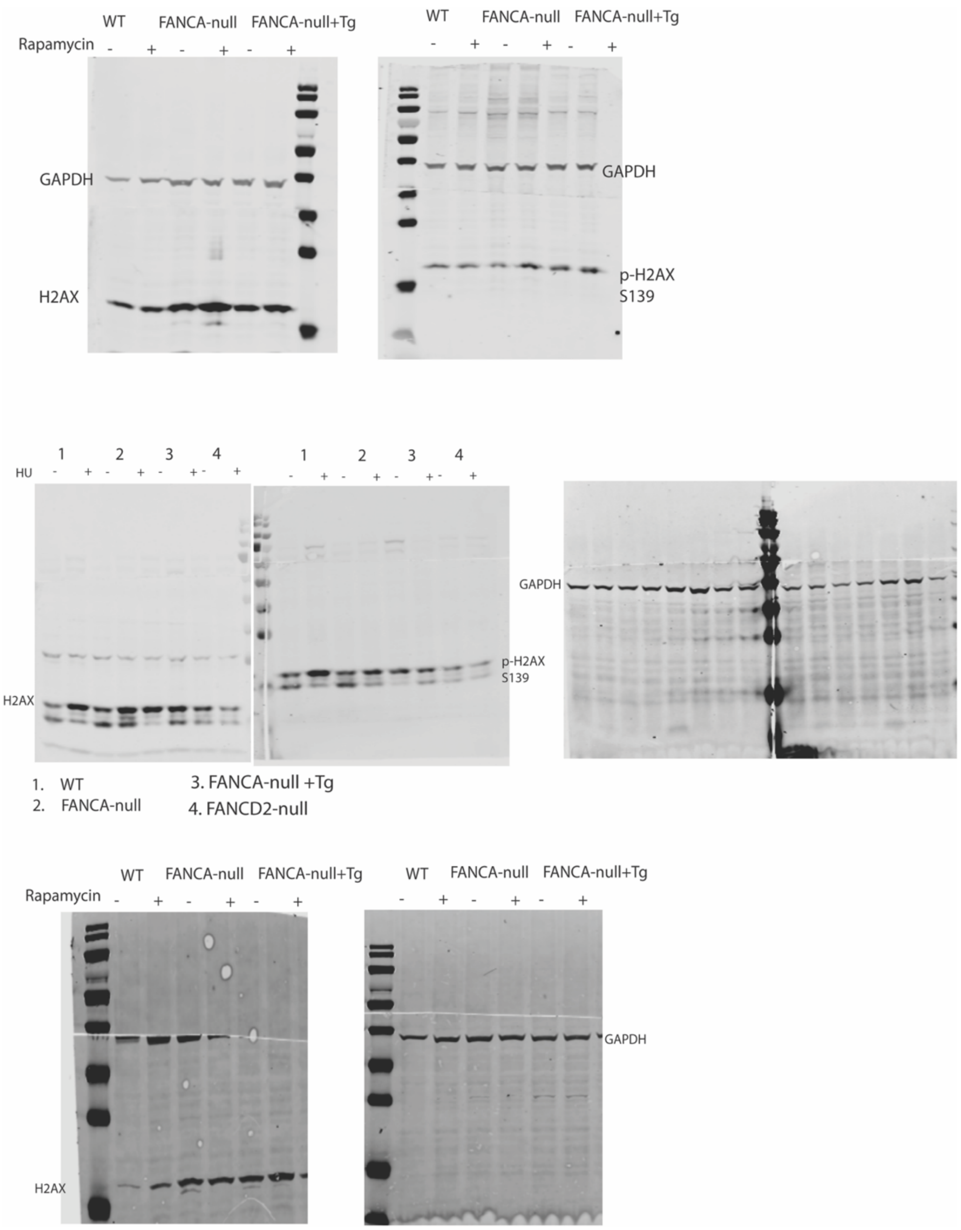
WT
FANCA-null
FANCA-null+Tg
Rapamycin
- + - + - +
GAPDH
H2AX
WT
FANCA-null
FANCA-null+Tg
- + - + - +
GAPDH
p-H2AX
S139
1 2 3 4
HU
- + - + - + - +
H2AX
1 2 3 4
- + - + - + - +
p-H2AX
S139
GAPDH
1. WT
2. FANCA-null
3. FANCA-null +Tg
4. FANCD2-null
WT
FANCA-null
FANCA-null+Tg
Rapamycin
- + - + - +
H2AX
WT
FANCA-null
FANCA-null+Tg
- + - + - +
GAPDH
